# Foreign Body in the Tongue for Thirty-two Years

**Published:** 1846-04

**Authors:** 


9. *Foreign Body in the Tongue for Thirty-two Years.*—A German soldier was wounded in the battle of Gross-Gorschen (2nd May, 1813) by a musket ball, which penetrated the left cheek, carrying away the four last molars of the upper jaw, and, passing through the tongue, made its exit through the left cheek, carrying away several teeth of the left side of the under jaw. The wounds healed in six weeks, and, except the loss of the teeth no other deformity remained but the cicatrix of the tongue, which did not impede his speaking or chewing. During the spring of the year, at which time the patient was subject to pulmonary and cerebral congestion, severe pains, with slight swelling of the tongue, came on, to which was added, in the year 1829, a small swelling of its right side, which suppurated and discharged thin matter, after which it gradually healed. On the 2d of May, 1845, a similar swelling made its appearance in the same place, which opened without discharging any matter, and after some days, what appeared to be a small piece of bone presented itself in the opening, which, on being removed, proved to be the second molar tooth, which had penetrated the tongue from the musket shot 32 years previously, and had during the whole time caused no great inconvenience. The roots of the tooth were broken off by the neck, and the whole surface covered by calcareous deposite.—*Oester Medecin. Wochenscrift*, in *Ibid.*

